# False Recognition in Short-Term Memory – Age-Differences in Confidence

**DOI:** 10.3389/fpsyg.2019.02785

**Published:** 2019-12-13

**Authors:** Barbara Sikora-Wachowicz, Koryna Lewandowska, Attila Keresztes, Markus Werkle-Bergner, Tadeusz Marek, Magdalena Fafrowicz

**Affiliations:** ^1^Department of Cognitive Neuroscience and Neuroergonomics, Institute of Applied Psychology, Jagiellonian University, Kraków, Poland; ^2^Center for Lifespan Psychology, Max Planck Institute for Human Development, Berlin, Germany; ^3^Brain Imaging Centre, Research Centre for Natural Sciences, Budapest, Hungary; ^4^Faculty of Education and Psychology, Eötvös Loránd University, Budapest, Hungary; ^5^Neurobiology Department, Neuroimaging Group, Malopolska Centre of Biotechnology, Jagiellonian University, Kraków, Poland

**Keywords:** visual short-term memory, age-related differences, false recognitions, confidence judgments, older adults

## Abstract

Compared to young adults, older adults are more susceptible to endorse false memories as genuine and exhibit higher confidence in their decisions to do so. While most studies to date have addressed this phenomenon in the context of episodic memory, the literature on age-differences in false recognition during short-term memory (STM) is scarce. Hence, the present study investigated age-related differences in the rate of false alarms (FA) and subsequent confidence judgments in STM. Thirty-three young and thirty-three older adults performed a visual short-term recognition memory task. In each trial, participants encoded a single abstract object, then made a “same” or “different” decision on a subsequent test, followed by a confidence judgment. We found significant age-related differences in performance as measured by the sensitivity index (*d*′), but not in the rate of FAs. Older adults were more confident in their erroneous recognition decisions than younger adults. The results are discussed in the context of age-differences in monitoring and associative processes.

## Introduction

Both older and younger adults may easily forget new material as well as falsely recall information that had never occurred (Gallo, 2010). However, older adults are in general more susceptible to memory errors and need more time to retrieve information (Reuter-Lorenz and Park, 2010; Devitt and Schacter, 2016). In the context of episodic memory, confidence accompanying memory decisions for false recognition of novel associations increases with age (e.g., Shing et al., 2009). This increased age-related susceptibility to high-confidence false alarms (FAs) has been primarily linked with impairments in binding and monitoring (Fandakova et al., 2013a, b). It is known that FAs are observed in STM (Atkins and Reuter-Lorenz, 2008; Abadie and Camos, 2019), but it is unclear whether age-differences in susceptibility to such errors also occur in STM and if yes, whether they are associated with age-differences in confidence. Thus, in the present study we aimed to investigate this phenomenon in a simple visual STM task.

### False Recognition in Younger and Older Adults

False memories, often defined as erroneous recognition of stimuli related to target memories, have been extensively studied in the context of episodic/long-term memory using paradigms based on conceptual and perceptual similarity (e.g., Ly et al., 2013; Pidgeon and Morcom, 2014), and with different types of target memories (e.g., Roediger and McDermott, 1995; Koutstaal et al., 1999). Three alternative but not mutually exclusive mechanisms have been offered to explain why we produce false memories: overreliance on gist or familiarity (e.g., Brainerd and Reyna, 2002), binding/associative deficits (e.g., Lyle et al., 2006), and impairments of source monitoring (see Mitchell and Johnson, 2010). Older adults were found to usually commit more FAs in episodic memory tasks than younger adults (for review see Devitt and Schacter, 2016).

False memories have been documented not only in episodic memory but also in STM for semantically related words (e.g., Deese, 1959; Roediger and McDermott, 1995; Atkins and Reuter-Lorenz, 2008, 2011; Abadie and Camos, 2019) as well as perceptually related objects (Lewandowska et al., 2018). The underlying mechanisms remain unclear with studies suggesting either common (Atkins and Reuter-Lorenz, 2008, 2011; Flegal et al., 2010) or complementary mechanisms of FAs in short- and long-term memory (Abadie and Camos, 2019). The recent findings of Abadie and Camos (2019) suggest that FAs in STM arise when verbatim memory (i.e., memory for detailed surface forms of items) no longer blocks gist long term memory (i.e., memory for general meaning or pattern, see Brainerd and Reyna, 2002). Accordingly, FAs in STM occur in tasks based on semantic-relatedness of words, in which verbatim memory can be easily impaired by either interference from multiple items or by a secondary task, and as a consequence, gist long-term memory can impact performance (Coane et al., 2007; Atkins and Reuter-Lorenz, 2008; Abadie and Camos, 2019). However, erroneous recognitions were observed also in tasks with abstract objects and visual masks (Lewandowska et al., 2018, 2019). In such tasks the influence of pre-existing semantic representations from long-term memory is reduced, and the verbatim memory is not affected by a secondary task (e.g., arithmetic distractor). Thus, in such procedures based on perceptual-relatedness FAs in STM cannot be easily explained by the influence of long-term memory. In consequence, rather common/partially common than complementary mechanisms of short- and long-term false memories can be assumed.

In contrast to episodic memory, the impact of age on false recognitions in STM is underexplored. While a number of studies have investigated age-related differences in visual short-term/working memory (e.g., Mitchell et al., 2000b; Peterson and Naveh-Benjamin, 2016; Rhodes et al., 2017), they did not focus on understanding false recognitions specifically. However, several lines of evidence suggest age-differences in false recognitions in STM. For instance, similarly to episodic memory, older adults are more prone to commit source errors in STM and working memory tasks (Hedden and Park, 2003; Mitchell and Johnson, 2010) and show deficits in inhibitory control of memory content (Gazzaley et al., 2005; Jost et al., 2011). Also, proactive interference from previously relevant content has a more robust negative impact on working memory of older than younger adults (Reuter-Lorenz and Park, 2010; Loosli et al., 2016).

Overall, such age-differences in STM performance seem to result from age-related impairments in the interplay of low-level feature binding and top-down control (e.g., Sander et al., 2011a, b; for review, see Sander et al., 2012). These binding and/or monitoring deficits may contribute to older adults’ higher rate of false recognitions in STM. For instance, Chen and Naveh-Benjamin (2012) showed that age-related differences in associative memory regarding processing complex stimuli (i.e., requiring binding of pairs of faces and scenes) derive from the number of FAs rather than hits – in both short-term/working and long-term memory in the continuous recognition task. Some recent studies, however, did not find clear evidence for older adults’ binding deficits in visual working memory (e.g., Peterson and Naveh-Benjamin, 2016; Brown et al., 2017; Rhodes et al., 2017).

Importantly, age-related changes observed in the hippocampus and prefrontal cortex, regions supporting binding and monitoring, respectively, have been implicated in age-related increases in FA in LTM (see Devitt and Schacter, 2016). Given at least partial overlap between neurocognitive mechanisms supporting episodic and STM functioning (Ranganath and Blumenfeld, 2005), including involvement of hippocampus in STM binding (e.g., Hannula et al., 2006; Yonelinas, 2013; Libby et al., 2014), these age-related changes are also expected to lead to FAs in STM. Hence, one might assume that in the context of STM, older adults would be more prone to commit FA errors than younger adults – similar to episodic memory.

### Age-Differences in Confidence Judgments

With regard to episodic memory, older adults have been shown to be more confident in their erroneous responses than younger adults (e.g., Jacoby and Rhodes, 2006; Shing et al., 2009; Wong et al., 2012; Fandakova et al., 2013a, b; Dodson et al., 2015). The current literature offers several possible (but not mutually exclusive) explanations for this phenomenon. Dodson et al. (2007a, b) have proposed that older adults may be prone to “misrecollections.” Here, during retrieval, older adults are assumed to erroneously re-combine features of different episodes into illusory “true” recollections. The miscombination account may also explain why older adults are more confident in their false memories compared to young adults: their false memories may be “true” recollections of features miscombined at encoding. Thus, these FAs are accompanied by high subjective confidence. At the same time age-differences in monitoring abilities have also been shown to contribute to older adults’ high-confidence FAs (Fandakova et al., 2013b).

Interestingly, the misrecollection account of older adults’ enhanced susceptibility to high confidence false recognitions resonates well with animal models of neurocognitive aging in the hippocampus (i.e., Wilson et al., 2006). Based on extensive analysis of rodent studies, it was proposed that aging is linked with a reduced ability to detect differences in inputs to the hippocampus (Wilson et al., 2006) – potentially resulting in miscombinations. Shing et al. (2011) extended this view on age-differences in associative memory performance in humans by demonstrating a direct link between older adults’ increased rate of FAs and specific reduction in hippocampal volume. Thus, older adults’ associative impairments can lead to generally higher rate of confident FAs.

Monitoring deficits have also been linked with a higher rate of confident FAs. The amount of such high confidence errors was negatively correlated with participants’ frontal lobe functioning as measured by the Wisconsin Card Sorting Test (Fandakova et al., 2013b), and in a continuous recognition task, younger adults presented an increase in prefrontal activity linked with increased monitoring demands, whereas older adults showed decreases in this region and declines in performance linked with increasing interference (Fandakova et al., 2014). Considering the crucial role of monitoring and top-down inhibitory control in efficient STM functioning (e.g., Gazzaley and Nobre, 2012), it should be assumed that age-related deficits in these abilities may contribute to older adults’ higher rate of highly confident FAs.

The goal of our study was to investigate age-differences in false recognitions and related confidence judgments in STM. In sum, theoretical considerations and empirical observations suggest common mechanisms for age-related changes in the generation of false recognition in episodic memory and STM. In particular, age-related associative and monitoring impairments may partly drive age-differences in FAs in both episodic memory and STM. Hence, when comparing younger and older adults’ performance in a visual STM task based on abstract objects, we expected higher FA rates for perceptually related lures in older adults. Moreover, due to impaired monitoring abilities (Fandakova et al., 2013b) and/or tendency to easily miscombine features (Dodson et al., 2007b), FAs in older adults were also assumed to be accompanied by higher confidence in the erroneous memory decisions, extending observations from episodic memory research (Dodson et al., 2007a; Shing et al., 2009; Fandakova et al., 2013b).

## Materials and Methods

### Participants

Sixty-six volunteers: 33 young (YA; *M*_age_ = 21.4 years, SD = 1.84, 29 females) and 33 older adults (OA; *M*_age_ = 60.2 years, SD = 4.54, 25 females) participated in the study. One older adult was excluded from all the analyses due to technical problems during data collection, and 2 YA and 2 OA were excluded as outliers, i.e., having FAs – or Hit-rates beyond inner fence from the group-specific median, defined as third quartile plus 1.5 times the interquartile range and first quartile minus 1.5 times the interquartile range. Hence, the final sample consisted of 31 younger (*M*_age_ = 21.45 years, SD_age_ = 1.89, 27 females) and 30 older adults *M*_age_ = 60.5 years, SD_age_ = 4.51, 22 females). All participants were without major health problems and had normal or corrected-to-normal vision. Younger participants were students recruited at the university, whereas older participants were recruited from the local community. All participants were familiarized with the procedure. This study was carried out in accordance with the recommendations of the APA Ethics Code. All subjects gave written informed consent in accordance with the Declaration of Helsinki. The study protocol was approved by the Committee for Research Ethics at the Institute of Applied Psychology at the Jagiellonian University.

### Experimental Task and Procedure

We used a recognition task with abstract objects and subsequent confidence judgments to measure STM performance. In order to ensure that STM is tested, we introduced single but rather complex, abstract (meaningless) items as targets, and used a visual mask instead of a distractor. The type of experimental material (single stimuli and masks) was chosen in order to preclude the possible influence of long-term memory, caused by (1) pre-existing semantic representations of the stimuli used, (2) the amount of material extending the capacity of individual’ STM, or (3) difficulties with active maintenance of the material (see Abadie and Camos, 2019). In addition, as participants were to memorize only one item and the distractor task was not introduced, it allowed for limiting the need for object manipulation typical rather for working memory than for simple short-term memory (STM) tasks (see Baddeley, 2012). Both time for recognition and for confidence judgment were selected based on response times reported in previous STM studies (e.g., Oberauer, 2005), and verified during pilots to ensure that older adults easily follow the procedure. The time restriction was also aimed to achieve a fairly consistent timing for a follow-up fMRI study.

Participants were presented with 120 trials, 40 of which included a target, 40 a stimulus similar to the target memory (lure), and 40 negative, clearly distinct stimulus (foil). Six versions of the task were created and counterbalanced across participants. Across the different versions, it was ensured that each stimulus was followed by each of the three trial types. In half of versions one of two similar objects served as a target, and in another half, the other one. Within each version the order of stimuli and trial types, as well as length of inter-stimulus and inter-trial intervals were randomized.

**FIGURE 1:**
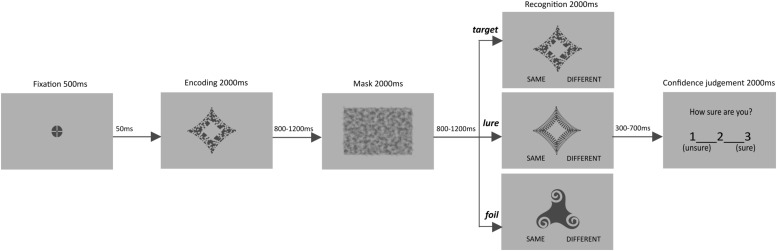
Schema of experimental task. Each trial was followed by ITI (2500–4500 ms).

In each trial, an item was presented for 2000 ms, followed by an inter-stimulus interval (ISI, 800–1200 ms, in steps of 100 ms), a visual mask (2000 ms), and a second ISI (800–1200 ms, in steps of 100 ms). Afterward, a memory test appeared for 2000 ms and participants were asked to determine whether the presented object is “same” or “different” compared to the to be memorized item. It was followed by an additional blank interval (300–700 ms, in steps of 100 ms), before the confidence question appeared: “How sure are you?” It was displayed for 2000 ms and participants were asked to rate confidence of their recognition response on a 3-point scale (from 1 – unsure to 3 – sure). Confidence judgments were followed by an inter-trial interval (ITI, 2500–4500 ms, in steps of 500 ms). Prior to the start of the next trial, a flashing fixation point appeared for 550 ms (see Figure 1).

The experimental task was presented with E-Prime 2.0 (Psychology Software Tools). All stimuli were shown at the center of the screen. The abstract objects occupied 6° 45′ of visual angle, whereas the visual mask subtended 14° 58′ × 11° 14′ of visual angle. Stimuli were presented in dark gray (RGB 72, 72, 72) on a light gray (RGB 176, 176, 176) background, made with Inkscape (GNU General Public License). The stimuli were previously used in similar STM tasks (Lewandowska et al., 2018, 2019). For additional examples of the stimuli, see Supplementary Figure 1). Overall, the task lasted for about 28 min and was preceded by instruction and demonstration of sample trials/training aimed to familiarize participants with the procedure.

## Results

Descriptive statistics of overall performance are given in Table 1. Trials with missing responses (e.g., participants pressed the wrong button or pressed the button too late) were excluded from the analysis. Data is presented after outliers’ exclusion.

**TABLE 1:** Overall performance for younger (YA) and older (OA) adults for “same” responses (i.e., Hits, False Alarms to Lures, and False Alarms to Foils), “different” responses (i.e., Misses, Correct Rejection of Lures, Correct Rejection of Foils), and missing responses for each trial type.

**Proportion of responses**	**Same responses**		**Different responses**		**Missing responses**	
**Trial type**	****YA****	****OA****	****YA****	****OA****	****YA****	****OA****
Positive	0.84 (0.02)	0.78 (0.03)	0.12 (0.02)	0.12 (0.03)	0.03 (0.01)	0.09 (0.02)
Lures	0.08 (0.01)	0.13 (0.01)	0.88 (0.01)	0.80 (0.02)	0.05 (0.01)	0.08 (0.01)
Foils	0.005 (0.002)	0.002 (0.002)	0.98 (0.01)	0.98 (0.01)	0.02 (0.01)	0.02 (0.01)

*Standard errors are shown in the brackets.*

### Age-Differences in STM Accuracy as Measured by the Sensitivity Index (*d*′), but Not Hit- or FA-Rate Alone

**FIGURE 2:**
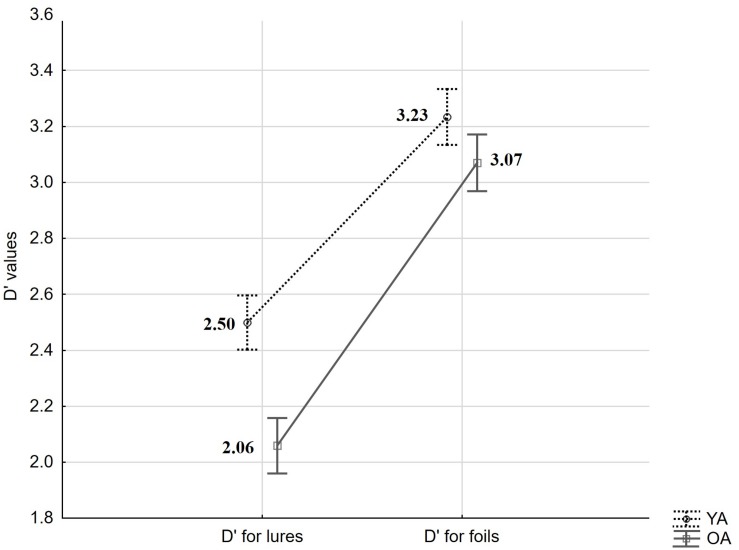
Mean d′ values derived from targets and lures and from targets and foils, by age groups (YA: young adults, OA: older adults). Error bars indicate standard errors.

In a first set of analyses, we asked whether accuracy differs between age-groups. Overall performance in the present task was assessed with a sensitivity index (*d*′) – an accuracy measure derived from Signal Detection Theory and calculated as *d*′ = *z(Hit) – z(FA)* (see Macmillan and Creelman, 2004). Here, Hit- and FA-rates were transformed by adding 0.5 to raw scores and dividing by *N* + 1, where *N* is the number of old or new trials, respectively (see Snodgrass and Corwin, 1988). A mixed measures ANOVA for *d*′ values with the within-person factor trial-type (FA to Lures vs. FA to Foils) and the between-person factor age (YA vs. OA) revealed a significant effect of trial type (*F*_(__1_,_59__)_ = 266.93, *p* < 0.001, ${\text{η}}_{\text{p}}^{2}$ = 0.82), a significant effect of age (*F*_(__1_,_59__)_ = 5.45, *p* = 0.02, ${\text{η}}_{\text{p}}^{2}$ = 0.08), and an interaction (*F*_(__1_,_59__)_ = 6.68, *p* = 0.01, ${\text{η}}_{\text{p}}^{2}$ = 0.10; see Figure 2). HSD Tukey *post hoc* revealed that age-differences result from changes in *d*′ based on FA to Lures (*p* = 0.01) but not Foils (*p* = 0.65).

A mixed measures ANOVA on accuracy with response type (Hit vs. FA to Lures, see Table 1) forming the within-person factor and age as a between-person factor (YA vs. OA) was conducted to examine the specific influence of age-differences on false and correct recognitions. Analyses revealed a significant effect of response type (*F*_(__1_,_59__)_ = 2100.28, *p* < 0.001, ${\text{η}}_{\text{p}}^{2}$ = 0.97) and an interaction effect (*F*_(__1_,_59__)_ = 13.79, *p* < 0.001, ${\text{η}}_{\text{p}}^{2}$ = 0.19), but not an effect of age (*F* = 0.06; *p* = 0.81). Importantly, HSD Tukey *post hoc* revealed that there were no significant age-differences either in FA-rates (*p* = 0.19) or in Hit-rates (*p* = 0.08). The interaction emerged because in each age group hit rate was higher than the false alarm rate in younger adults as well as older adults (all *p*s < 0.001).

In sum, we observed a significant difference between *d*′ values calculated with the use of FA to Lures and FA to Foils, showing that our procedure was generally effective in eliciting false recognitions based on perceptual similarity. We observed age-differences in STM accuracy as measured by item-specific *d*′ involving Hits and FA to Lures (but not to Foils). However, in contrast to our expectations, this effect was not significantly related to older adults’ higher FA rate. Rather, it was built up by non-significant age-differences in performance on both positive and lure trials.

### Older Adults Have Higher Confidence in Their False Memory Decisions

Next, we asked whether age-differences in memory confidence would point to differential monitoring abilities between the two age groups. Importantly, younger and older adults used the confidence scale similarly: There were no age-specific differences in confidence distribution, and ISI did not affect age-differences in confidence after errors (see Supplementary Material).

First, the ability to adjust subjective level of confidence after errors was assessed with the metacognitive sensitivity index – *type two d*′, calculated as *type two d*′ = *z(type two Hit) – z(type two FA)*, where *type two Hit* stands for high-confidence correct responses as a proportion of all correct responses for both targets and lures (i.e., hits and correct rejections), and *type two FA* stands for high-confidence incorrect responses as a proportion of all erroneous responses for both targets and lures (i.e., misses and false alarms; see Fleming and Lau, 2014). While *d*′ indicates the participants’ abilities to discriminate old and new stimuli, *type two d*′ indicates participants’ general ability to effectively monitor their performance and adjust the confidence level regarding the responses accuracy. Similar to type one *d*′, type two Hit- and type two FA-rates were transformed by adding 0.5 to raw scores and dividing by *N* + 1 (see Snodgrass and Corwin, 1988). An unpaired *t*-test for *type two d*′ values of YA and OA revealed significant age-differences (*t(59)* = 5.24, *p* < 0.001), with older adults presenting lower metacognitive sensitivity (*type two d*′ = 0.83, SE = 0.10) than younger adults (*type two d*′ = 1.55, SE = 0.09).

**FIGURE 3:**
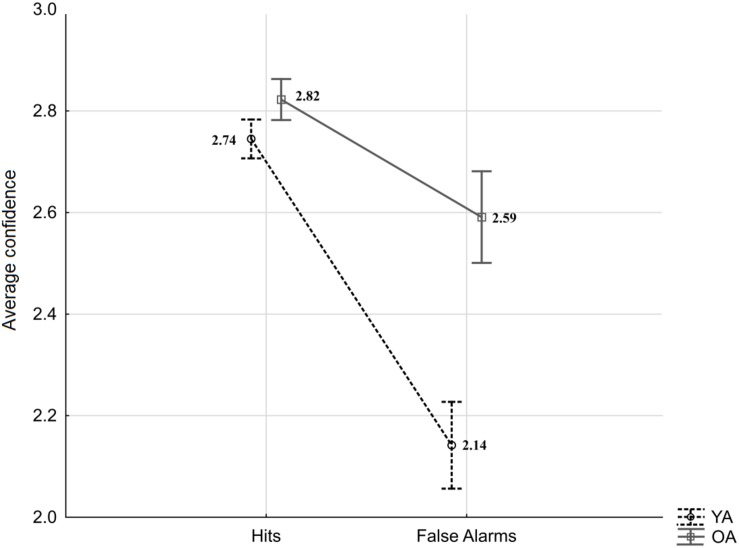
Average confidence for Hits and False Alarms by age groups (YA: young adults, OA: older adults). Error bars indicate standard errors.

As indicated by the equation, *type two d*′ takes into account correct and incorrect responses for both targets and lures, and as such it does not allow for a more specific comparison of younger and older adults’ confidence after true recognitions (“same” responses to targets; Hit) and false recognitions (“same” responses to lures; FA). Therefore, the separate analysis was conducted to exclusively address age-differences in “same” responses and verify whether they are observed in confidence judgments following hits, FA or both. The averaged confidence ratings for type one Hit and FA to Lures were subjected to a mixed-measures ANOVA with the within-person factor response type (Hit vs. FA) and the between-person factor age (YA vs. OA). The results revealed significant effects of age (*F*_(__1_,_53__)_ = 10.88, *p* = 0.002, ${\text{η}}_{\text{p}}^{2}$ = 0.17), response type (*F*_(__1_,_53__)_ = 60.18, *p* < 0.001, ${\text{η}}_{\text{p}}^{2}$ = 0.53), as well as an interaction effect (*F*_(__1_,_53__)_ = 11.94, *p* = 0.001, ${\text{η}}_{\text{p}}^{2}$ = 0.18; see Figure 3). HSD Tukey *post hoc* revealed that the average confidence for FA responses committed by younger adults was significantly lower than confidence for FA in older adults (*p* < 0.001). In addition, confidence in FA response was lower compared to Hit responses in younger adults (*p* < 0.001), as well as in older adults (*p* = 0.02). The confidence in Hit responses was comparable between age groups (*p* = 0.86).

In sum, the analysis of confidence judgments shows age-differences: Compared to younger participants, older adults demonstrated generally poorer metacognitive abilities and, more specifically, higher confidence in their erroneous acceptance of lure items as target memories.

### Response-Type Specific Age Differences in Confidence Judgment Reaction Times

Next, we asked whether response-type specific age-differences in reaction times (RT) could explain the observed higher confidence in FA responses in older adults. Typically, fast RTs indicate strong evidence for a given response alternative (e.g., Navon, 1975; Weidemann and Kahana, 2016). Hence, prolonged RT for FA can reflect the increased need for monitoring the outcome of a retrieval attempt, to support evidence accumulation. In addition, more time may also be necessary to recruit cognitive processes to overcome interference (e.g., Stroop, 1992). While older adults’ present generally longer RTs, this age-difference can be disproportionately larger, e.g., when correctly rejecting familiar items (Oberauer, 2005). Therefore, we analyzed age-differences in RTs of both recognition and confidence decisions to identify potential mechanisms underlying highly confident FA in OA in our task. Due to the low amount of low-confidence responses, overall mean RT was computed across all confidence-levels.

**FIGURE 4:**
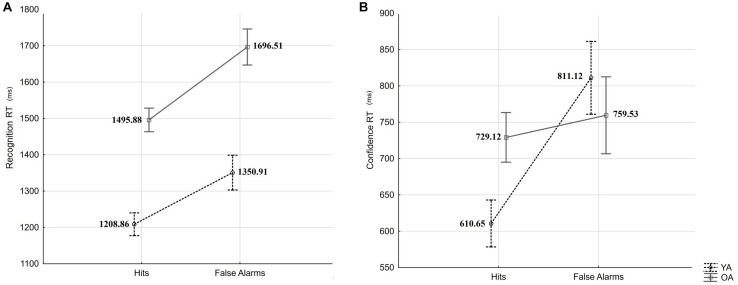
Recognition RTs **(A)** and confidence RTs **(B)** for Hits and False Alarms by age groups (YA: young adults, OA: older adults). Error bars indicate standard errors.

In a first analysis, the recognition RT was subjected to a mixed-measures ANOVA with the within-person factor response type (Hits vs. FAs) and the between-person factor age (YA vs. OA). The results revealed a significant effect of response type (*F*_(__1_,_54__)_ = 36.14, *p* < 0.001, ${\text{η}}_{\text{p}}^{2}$ = 0.40) and a significant effect of age (*F*_(__1_,_54__)_ = 38.79, *p* < 0.001, ${\text{η}}_{\text{p}}^{2}$ = 0.42), but not an interaction effect (*F* = 1.06, *p* = 0.31; see Figure 4A). Older adults presented slower responses than younger adults, and both groups presented slower reactions for FA than Hits, indicating that age-differences in confidence observed in our experiment are not likely to be explained by age-differences in speed-accuracy trade-off.

In a second analysis, confidence RT was subjected to mixed-measures ANOVA with the within-person factor response type (Hits vs. FA) and the between-person factor age (YA vs. OA). The results revealed a significant main effect of response type (*F*_(__1_,_53__)_ = 16.24, *p* < 0.001, ${\text{η}}_{\text{p}}^{2}$ = 0.23) and an interaction effect (*F*_(__1_,_53__)_ = 8.81, *p* = 0.004, ${\text{η}}_{\text{p}}^{2}$ = 0.14), but no main effect of age (*F* = 0.38, *p* = 0.54; see Figure 4B). HSD Tukey *post hoc* revealed that confidence judgments RTs differed significantly between Hits and FAs for younger adults (*p* < 0.001), but not for older adults (*p* = 0.88). We have observed no significant age-differences in confidence RT after Hits (*p* = 0.24), and after FAs (*p* = 0.86).

In sum, we did not observe response-type specific age-differences in recognition RT but observed an age × response type interaction in the RT of confidence judgments. The fact that, compared to Hits, in both age groups, recognition RTs were slower for FAs suggests calibrated engagement of monitoring and/or associative processes in both age groups at retrieval. However, the fact that only younger adults showed the same RT pattern (slower RTs for FA compared to Hits) in confidence judgments suggests greater engagement and/or effectiveness of monitoring process in young compared to older adults.

## Discussion

In this study, we tested whether the pattern of age-related differences in FAs in item-based visual STM is comparable to that observed in episodic memory. In contrast to previous observations in episodic memory (for review see Devitt and Schacter, 2016), we did not find evidence supporting the hypothesis that older adults are more likely to commit FAs in STM compared to young adults. However, consistent with prior findings on episodic memory, we did find that older adults were more confident in their false memories than younger adults. The obtained results show that age-related differences in confidence after false recognitions in STM may persist despite attenuated age-differences in FA rate. Below, we discuss the main findings in detail.

### Older Adults’ Lower STM Performance Is Not Driven by Their Significantly Greater Susceptibility to False Recognitions

Our procedure was successful in eliciting similarity-based false memories a few seconds after encoding (similarly to Coane et al., 2007; Atkins and Reuter-Lorenz, 2008, 2011; Lewandowska et al., 2018, 2019), as indicated by significantly lower d′ values for perceptually related lures than for unrelated foils. We also observed age-differences in participants’ ability to discriminate targets from lures but not from foils. However, contrary to our hypothesis, age-differences in accuracy levels in our STM task were not specifically related to FAs (neither age-difference in hit rate nor in FAs’ rate were significant). Instead, they resulted from a more general inability to discriminate targets from lures.

The observed inconsistency between our results and the ones from episodic memory can be associated with impact of feature binding on age-related changes in working memory (see Peterson and Naveh-Benjamin, 2016; Brown et al., 2017; Rhodes et al., 2017). Whereas many researchers observe age-related differences in associative deficits over short period of time (e.g., Chen and Naveh-Benjamin, 2012), the results seem to depend on many factors such as a presence of concurrent load or the type of binding required (see Peterson and Naveh-Benjamin, 2016). For instance, the ability to remember an object’s surface features and its combinations was reported to remain relatively intact with age (e.g., Rhodes et al., 2016). The study of Brown et al. (2017) also indicated that visual feature binding in STM may be relatively resistant to changes with age, with both age groups being affected similarly. Yet, they noted the possibility that there is a small effect of age, and its statistical significance may depend on used methodological approach. Being a step further, Rhodes et al. (2017) argue against age-specific differences in binding deficits in visual STM. Relatedly, older adults’ higher FA rates have been directly linked to their associative deficits (e.g., Naveh-Benjamin, 2000). Thus, the fact that, contrary to our expectations, we did not observe significant age-differences in FAs may result from an attenuated effect of age on binding abilities in visual STM.

The properties of an experimental design, including number of objects used, could have also contributed to the diminished age-differences in FA rate. Previous studies suggest age-related increases in FAs being more robust when instead of single items, multiple images (in episodic memory, Pidgeon and Morcom, 2014) or a combination of features (in STM, Mitchell et al., 2000a, b) had to be memorized. Accordingly, hippocampal activity, linked with age-related differences in binding in episodic memory, in working memory and perception has been observed when the material was sufficiently complex and high-resolution associations between features were required (Yonelinas, 2013). Also, studies on patients with amnesia showed that, at short lags, memory for relations/conjunctions is impaired more than memory for items or locations alone (Hannula et al., 2006; Olson et al., 2006). Hence, even though complex objects with multiple features were used, the potentially lower associative demands of our item-based STM task may also contribute to the attenuated age-differences in performance.

Alternatively, the type of experimental material could also affect the age-differences in susceptibility to false recognitions, as in long-term memory they were found to be lower when pictures were used instead of words (Smith et al., 2015), or when abstract shapes were used instead of concrete objects (Koutstaal et al., 2003; Pidgeon and Morcom, 2014). These observations suggest that older adults may be less sensitive to perceptual than conceptual relatedness (see Koutstaal et al., 2003; Pidgeon and Morcom, 2014).

Additionally, speeded responding, resulting from the restricted response time window, could have impacted age-differences in performance, e.g., by influencing group differences in speed-accuracy tradeoffs (e.g., Forstmann et al., 2011). Also, restricted time tends to inflate the influence of familiarity during recognition decisions (Yonelinas, 2002 for review). In turn, familiarity would increase false alarm rate. However, in our task not only was the rate of FAs comparable between age groups, but we also did not observe age-specific differences in recognition RT (see the “Results” section) nor age-differences in rate of missing responses on lure probes (see Table 1 in the “Results” section). In addition, participants’ tendency to make “same” or “different” responses for lures and targets (i.e., decision criterion, see Macmillan and Creelman, 2004) was comparable between groups (see Supplementary Material). Besides, given that older adults are slower (e.g., Reuter-Lorenz and Park, 2010), increasing time for responding would rather improve older adults’ performance. Taken together, restricted response time was not likely to influence the attenuated FA rate in older adults. The other possible explanation is that age-differences in FA occur later in adulthood. In order to test all the previously mentioned alternative explanations, future studies explicitly manipulating type and number of stimuli, time for response, as well as participants’ age are needed. Here, we aimed to determine whether age-differences in FAs and following confidence occur in simple item-based STM task, which does not involve activation of pre-existing semantic representations from long-term memory and limits the influence of other processes such as objects manipulation or suppression of unimportant information.

Finally, it is important to note that older adults presented diminished ability to discriminate between targets and lures, as indicated by the sensitivity index. Target-lure discrimination could have been more difficult for older adults compared to younger, due to indistinctive encoding and reduced retrieval of details (Dennis et al., 2014; McDonough et al., 2014). The observed age-differences in performance could be also driven by changes in executive functioning (e.g., Gazzaley and Nobre, 2012; Sander et al., 2012). Older adults often exhibit diminished executive top-down control and less effective monitoring (Gazzaley et al., 2005; Fandakova et al., 2014). Considering their important role in STM (e.g., Gazzaley and Nobre, 2012), we propose that age-differences in executive functioning could, together with reduced or distorted retrieval of details, contribute to the observed differences in target-lure discrimination.

In sum, older adults’ decline in sensitivity observed in the present visual STM task was not underlaid by significant age-differences in FA rates. It most likely resulted from attenuated age-differences in visual STM binding (e.g., Brown et al., 2017) and/or the fact that only one abstract shape had to be memorized and as such associative/binding demands of the task were attenuated. Hence, the observed differences in the sensitivity index are likely to result from age-differences in executive functioning, together with older adults’ reduced encoding and/or retrieval of details rather than only their susceptibility for miscombinations.

### Monitoring Impairments Contribute to Older Adults’ Higher Confidence in False Memory Decisions

Despite attenuated age-differences in the number of false recognitions, older adults were more confident of their FAs than younger adults. Age-differences in memory calibration abilities have previously been observed in episodic memory (Jacoby and Rhodes, 2006; Shing et al., 2009; Wong et al., 2012; Fandakova et al., 2013b; Dodson et al., 2015). In line with these findings, in our task older adults’ impaired abilities to assess confidence (as indicated by their generally higher confidence and lower *type 2 d*′) were linked to age-differences in confidence after errors rather than hits (see also Fandakova et al., 2013b). Also, similarly to the previous studies (e.g., Dodson et al., 2007a), older adults’ higher confidence in FAs was not coupled with age-specific differences in overall distribution of confidence judgments – both age groups used the confidence scale in a similar manner with the highest number of “sure” responses and the lowest of “unsure” (see Supplementary Table 1). Taken together, it indicates that also in STM older adults lower their confidence after errors to a lesser extent than younger adults do.

In the context of episodic memory, older adults’ highly confident FAs have been linked to both associative (Dodson et al., 2007a; Shing et al., 2009) and monitoring impairments (Fandakova et al., 2013a, b). According to the misrecollection account (Dodson et al., 2007b, 2015), older adults’ hyperactive binding leads to “true” false memories, i.e., to illusory recollection of miscombined features. However, if older adults’ higher confidence after FAs in STM would be primarily a consequence of older adults’ higher susceptibility to misrecollections (Dodson et al., 2007a), it should result both in higher FAs rate and higher confidence. Instead, we did not find evidence that age-differences in sensitivity in our task were driven by older adults’ susceptibility to false recognitions. It suggests that some additional processes underlie persisting age-differences in confidence. Accordingly, Flegal and Reuter-Lorenz (2014) indicated that unlike the rate of FAs, the confidence judgments are not influenced by processing depth and remain stable across short and long delay, perhaps depending rather on monitoring failures. Consistently, observed age-differences in type two sensitivity index further suggest that age-differences in monitoring contributed to older adults’ less adequate confidence assessment, as this more general measure includes both highly confident FAs as well as misses for targets.

Thus, although the overall pattern of results did not preclude some influence of miscombinations, it consequently suggests contribution of monitoring deficits to older adults’ highly confident errors. Importantly, in the context of episodic memory age-differences in susceptibility to high-confidence FAs have been already associated with age-related impairments in monitoring and strategic processes (Shing et al., 2008; Wong et al., 2012; Fandakova et al., 2013a, b). For instance, it was shown (Fandakova et al., 2013b) that, irrespective of type/source of FAs, older adults were more confident of their FAs, and the tendency of participants to commit high-confidence errors was associated with poorer performance on Wisconsin Card Sorting Test. These results suggest that age-related increases in high-confidence FAs may be in part the result of impairments in prefrontally driven monitoring processes rather than associative deficits.

As top-down control and strategic monitoring, crucial for effective working memory functioning (Edin et al., 2009; Gazzaley and Nobre, 2012; Sander et al., 2012), show impairments with aging (e.g., Sander et al., 2012), the between-groups differences in memory calibration in our task may result from age-related declines in efficiency of these processes. Accordingly, older adults seem to present deficits in adjusting their monitoring involvement in more challenging, error-prone situations (Paige et al., 2016; Fandakova et al., 2018). Besides, studies suggest that in episodic memory older adults’ reduced retrieval of perceptual details is accompanied by increased prefrontal monitoring during correct recognitions but not FAs (Dennis et al., 2014). While both monitoring processes and memory representations are impaired, the reliability of subjective memory judgments can be diminished. For instance, older adults’ overreliance on familiarity-based monitoring can be strengthened (see Yonelinas, 2002; Devitt and Schacter, 2016), leading to overconfidence during FAs. This can be further enhanced by the time pressure during responding.

Analysis of RTs also suggests that in STM, older adults’ high-confidence judgments are partly rooted in monitoring impairments. Both in younger and older adults’ hits were processed faster than FAs. We did not find age-modulation in RT during recognition test, thus, the observed age-differences in confidence for FAs are unlikely to be explained by different information processing strategies, e.g., differences in speed-accuracy trade-off (see Oberauer, 2005). Noteworthy, older adults disproportionately longer RT for lures was previously linked with their deficits in binding (Oberauer, 2005), thus we did not find support for age-specific associative impairments which could impact older adults’ confidence selectively after FAs.

In addition, we found age-specific differences in time of confidence judgment – younger adults made faster confidence judgments after hits than after errors, whereas this difference was not observed for older adults. Again, it supports the conclusion that older adults’ high-confidence judgments are the result of diminished and/or ineffective monitoring and cognitive control, which cannot effectively compensate reduced retrieval of perceptual details.

In sum, in our STM task, we observed older adults’ less efficient memory calibration and their higher confidence of false recognitions. These effects seem to be at least partially driven by the age-related monitoring deficits.

## Conclusion

In the present STM task, older adults performed worse than younger adults, and age-related differences were observed in the level of confidence after errors. Unexpectedly, age-differences in performance level were not driven by older adults’ greater susceptibility to false recognitions. Previous work linked older adults’ tendency to commit more FAs with impaired associative binding abilities (e.g., Shing et al., 2011). The lack of significant between-groups differences in false recognitions in our item-based task most likely results from diminished age- differences in binding expressed in our visual STM task (see e.g., Brown et al., 2017). However, as performance in STM tasks strongly depends on executive functioning (Gazzaley et al., 2005; Sander et al., 2012), we suggest that the observed age-related differences in performance as measured by the sensitivity index, and in confidence level may reflect older adults’ deficits in monitoring abilities and their reduced encoding and/or retrieval of details rather than misrecollections alone. The fact that both age groups presented higher RTs during FAs than during hits is in line with this assumption, suggesting that age-differences in confidence are unlikely to be the result of different strategies of processing information at retrieval (see Oberauer, 2005).

These results provide further support for the contribution of older adults’ monitoring impairments to their tendency to commit high confidence FAs, as it was already indicated by Fandakova et al. (2013b) in the context of episodic memory. In addition, we demonstrated that age-related differences in confidence levels may be observed even when differences in performance are not driven by false recognitions of lures. Taken together, our experiment provided some insight into the nature of age-differences in high confidence FAs and broaden the knowledge of older adults’ confident errors in STM domain. While the visual item-based STM procedure seems promising to disentangle influence of associative and monitoring processes on confident FAs, future studies manipulating properties of the task, such as the number of stimuli and the time for responding, are needed. Also, future experiments should include measures of neural activity, e.g., fMRI, which could provide further insights into neuro-cognitive processes underlying age-differences in confident false recognitions in STM and help to better separate the contributions of associative and monitoring mechanisms.

## Data Availability Statement

The datasets generated for this study are available on request to the corresponding author.

## Ethics Statement

The studies involving human participants were reviewed and approved by the Committee for Research Ethics, Institute of Applied Psychology, Jagiellonian University, Kraków, Poland. The patients/participants provided their written informed consent to participate in this study.

## Author Contributions

BS-W, KL, AK, MW-B, TM, and MF: conceptualization, methodology, and writing – reviewing and edition. BS-W: data curation, formal analysis, investigation, and writing – original draft preparation. BS-W and MF: funding acquisition. AK, MW-B, and TM: supervision.

## Conflict of Interest

The authors declare that the research was conducted in the absence of any commercial or financial relationships that could be construed as a potential conflict of interest.
