# Supplementary material for: False Recognition in Short-Term Memory – Age-Differences in Confidence
Source: Front Psychol. 2019 Dec 13;10:2785. doi: 10.3389/fpsyg.2019.02785 (PMC6923284; doi:10.3389/fpsyg.2019.02785)
Supplement: Supplementary file 1 [file Table_1.docx]

Supplementary Material

# Examples of experimental stimuli


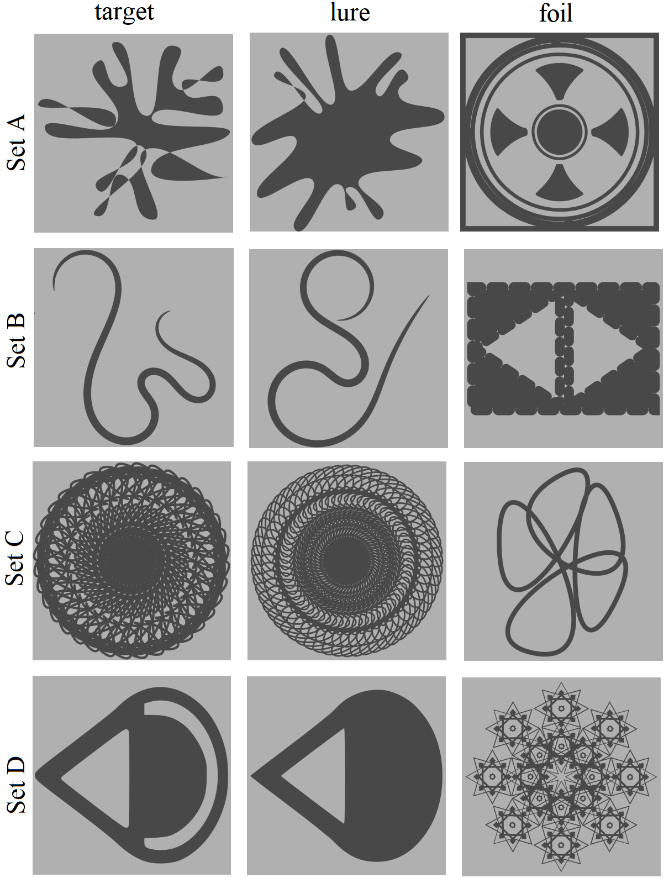


Supplementary Figure 1. Examples of experimental stimuli (four different sets).

# No correlations between timing of the task and confidence of FAs

There is no significant correlation between average confidence after FA and average time from memoranda to erroneously recognized stimuli (YA: 0.16, *p*=0.41, OA: 0.29, *p*=0.15) nor between average confidence after FA and average time from erroneously recognized stimuli to confidence question (YA: -0.02, *p* =0.92, OA: -0.28, *p*=0.17).

# Comparable confidence distribution in younger and older adults

Rates of different type of confidence responses across task (see Supplementary Table 1) were subjected to a mixed-measures ANOVA with confidence response as the within-person factor (sure, semi-sure, unsure) and age as the between-person factor (YA vs. OA). The results revealed significant effects of confidence response (*F*_(2,118)_ = 1569.82, *p* <0.001, *η_p_^2^* = 0.96), and an effect of age (*F*_(1,59)_ = 11.94, *p* = 0.001, *η_p_^2^* = 0.17), but no interaction effect (*F*_(2,108)_ = 0.26, *p*=0.77, *η_p_^2^* = 0.004). This indicates that both age-groups used confidence scale in a similar manner.

| Group | "sure" (3) | "semi-sure" (2) | "unsure" (1) |
| --- | --- | --- | --- |
| YA | 0.83 *(0.02)* | 0.12 *(0.01)* | 0.04 *(0.01)* |
| OA | 0.82 *(0.02)* | 0.09 *(0.02)* | 0.02 *(0.01)* |

**Supplementary Table 1. Overall confidence distribution for younger (YA) and older (OA) adults.** Standard errors are shown in the brackets.

# Rates of missing responses

An unpaired t-test for rate of missing responses for lures (see Table 1 in the main text) revealed no significant age-differences (*t(59)* = -1.85, *p* = 0.07). Similarly, an unpaired t-test for rate of missing responses for foils (see Table 1 in the main text) revealed no significant age-differences (*t(59)* = -0.18, *p* = 0.86). An unpaired t-test for rate of missing responses for targets (see Table 1 in the main text) revealed significant age-differences (*t(59)* = -3.40, *p* = 0.001), with older adults presenting more missing responses than younger adults.

# Decision criterion

The decision criterion – a bias measure derived from Signal Detection Theory and calculated as *c=-0.5*(z(Hit) + z(FA))* (see Macmillan and Creelman, 2004). Same as in *d’* analyses, Hit- and FA-rates were transformed by adding 0.5 to raw scores and dividing by N+1, where N is the number of old or new trials, respectively (see Snodgrass and Corwin, 1988). An unpaired t-test for decision criterion derived from lures and targets revealed no significant age-differences (*t(59) =* 0.14, *p*=0.89), showing that younger and older adults did not differ in their tendency to decide whether stimulus is “same” or “different” (YA M = 0.18, SE = 0.05; OA M = 0.17, SE = 0.08).
